# Peer Review of “Data Obfuscation Through Latent Space Projection for Privacy-Preserving AI Governance: Case Studies in Medical Diagnosis and Finance Fraud Detection”

**DOI:** 10.2196/72525

**Published:** 2025-03-12

**Authors:** Trutz Bommhardt

**Affiliations:** 1 University of Wuppertal, Wuppertal, Germany

**Keywords:** privacy-preserving AI, latent space projection, data obfuscation, AI Governance, machine learning privacy, differential privacy, k-anonymity, HIPAA, GDPR, compliance, data utility, privacy-utility trade-off, responsible AI, medical imaging privacy, secure data sharing, LSP, artificial intelligence

*This is a peer-review report for “Data Obfuscation Through Latent Space Projection for Privacy-Preserving AI Governance: Case Studies in Medical Diagnosis and Finance Fraud Detection.”*

## Round 1 Review

### General Comments

I thoroughly enjoyed reading this paper [1] as it is a well-written article that will make an important contribution to the literature on the development of privacy-preserving artificial intelligence (AI) governance. I have attached a few comments to improve the study.

### Specific Comments

#### Major Comments

Something like a discussion that embeds the latent space projection for AI governance and the results in the current scientific debate is missing before or after Chapter VII.

#### Minor Comments

In Chapter II B (Existing privacy-preserving techniques), please provide some further sources to demonstrate that the challenges mentioned are still relevant, as some sources are relatively old (eg, from 2009).

## Abbreviations

AI: artificial intelligence
